# Pathological gamblers are more vulnerable to the illusion of control in a standard associative learning task

**DOI:** 10.3389/fpsyg.2013.00306

**Published:** 2013-06-17

**Authors:** Cristina Orgaz, Ana Estévez, Helena Matute

**Affiliations:** ^1^Department of Psychology, Universidad Nacional de Educación a DistanciaMadrid, Spain; ^2^Department of Psychology, Universidad de DeustoBilbao, Spain

**Keywords:** gambling, illusion of control, associative learning, contingency learning, contingency judgments, causal learning

## Abstract

An illusion of control is said to occur when a person believes that he or she controls an outcome that is uncontrollable. Pathological gambling has often been related to an illusion of control, but the assessment of the illusion has generally used introspective methods in domain-specific (i.e., gambling) situations. The illusion of control of pathological gamblers, however, could be a more general problem, affecting other aspects of their daily life. Thus, we tested them using a standard associative learning task which is known to produce illusions of control in most people under certain conditions. The results showed that the illusion was significantly stronger in pathological gamblers than in a control undiagnosed sample. This suggests (1) that the experimental tasks used in basic associative learning research could be used to detect illusions of control in gamblers in a more indirect way, as compared to introspective and domain-specific questionnaires; and (2), that in addition to gambling-specific problems, pathological gamblers may have a higher-than-normal illusion of control in their daily life.

The perception of control over important events in our lives has been studied from many different perspectives in psychology. It allows us to predict the consequences of our actions and the actions of others, which adaptively can imply the difference between surviving and perishing. Sometimes, however, perceived control is not real. People often fail to distinguish those events that are controllable from those that are not, which gives rise to the illusion of control (Langer, 1975). The illusion of control can be defined as the tendency to believe that our behavior is the cause of the occurrence of desired events that occur independently of our own actions (Alloy and Abramson, 1979; Taylor and Brown, 1988; Matute, 1996).

The illusion of control is a universal phenomenon which has been observed to occur in most people and under many different conditions. Many laboratory experiments have shown that college students develop the illusion that they are controlling uncontrollable lights or tones or lottery tickets (e.g., Langer, 1975; Alloy and Abramson, 1979; Wasserman et al., 1983; Matute, 1996; Aeschleman et al., 2003; Msetfi et al., 2005). Illusions of control have also been reported in students trying to cure fictitious patients in a medical decision task (Blanco et al., 2011), or in Internet users who are trying to obtain points in an otherwise uncontrollable computer game (Matute et al., 2007). The illusion of control is also well-known among athletes and sports players, who often feel that a given ritual or lucky charm is necessary for success (Bleak and Frederick, 1998), or even in sport spectators, who tend to feel that supporting (or not) their favorite team through their TV at home contributes to the happy (or disastrous) score of the team (Pronin et al., 2006). Trading and consumer behavior have also been shown to be vulnerable to the illusion of control (Fenton-O'Creevy et al., 2003; Kramer and Block, 2011), as have companies and organizations themselves (Durand, 2003).

Finding out which conditions modulate the development and maintenance of the illusion of control is therefore important, given that it affects almost anyone and almost any decision or aspect in our daily life. Thus, at the same time that there is an extensive scientific literature which has highlighted the universality of this bias, there is also an important research agenda which explores the degree to which the illusion of control is sensitive to individual differences among humans.

The study of individual differences in the illusion of control has been concerned with gender (with women generally showing stronger illusions of control than men; see Alloy and Abramson, 1979; Wong, 1982; Vyse, 1997; Wolfradt, 1997; Dag, 1999), superstitious attitudes (Rudski, 2004), psychopathology (e.g., Wolfradt, 1997; Dag, 1999); cooperative behavior (Morris et al., 1998; Goldberg et al., 2005), or even sports (Laurendeau, 2006). It is also possible to come across studies about the illusion of control in psychological disorders such as depression (with depressed people generally being less vulnerable to the illusion of control; see Alloy and Abramson, 1979; Vázquez, 1987; Blanco et al., 2009, 2012), obsessive-compulsive disorder (Reuven-Magril and Reuven, 2008) and physical health (Harris and Middelton, 1994).

Pathological gambling is one of several psychological disorders with which the illusion of control has been most strongly associated (Ladouceur et al., 1984; Wolfgang et al., 1984; Coventry and Norman, 1998; Källmén et al., 2008; Lingyuan and Austin, 2008). It is a disorder of impulse control in which cognitive distortions are assumed to play an important role (Myrseth et al., 2010). According to some researchers (Sharpe, 2008; Lund, 2011) people with pathological gambling disorder bet because they hold wrong or irrational beliefs about the game and their ability to influence its outcome. Pathological gambling is closely related to the perception of the player that, to some extent, he or she can control the outcome of his or her bets (Goodie, 2005).

According to some, however, the lack of valid measures has impeded the systematic investigation of cognitive biases in gamblers (MacKillop et al., 2006). Data suggestive of an illusion of control in gamblers have often been obtained through talk-aloud methods and self-reporting measures and almost always in domain-specific (i.e., gambling) conditions (e.g., Dickerson, 1993; Strickland et al., 2006). As is already well-known in the literature, this type of data collection can be subject to a series of social desirability biases, avoidance of cognitive dissonance, or even investigator biases, particularly when the questions are related to the variable under study (i.e., in this case, gambling). Recent reviews have shown that the contribution of cognitive distortions to the development and maintenance of pathological gambling behavior is still in need of further scrutiny (Fortune and Goodie, 2011). Furthermore, some researchers from the clinical domain have argued, against the view of many others (e.g., Coventry and Norman, 1998; Källmén et al., 2008; Hudgens-Haney et al., 2013), that the illusion of control has only a limited influence in the maintenance of gambling behavior (Labrador et al., 2002; Mañoso et al., 2004). A better understanding of gamblers' subjective judgments of control seems therefore a necessary step in clarifying the etiology and maintenance of pathological gambling behavior (Matheson et al., 2009).

A question of particular interest is whether pathological gamblers actually suffer from a general distortion in their perception of control or is, by contrast, a domain-specific problem what they suffer. If it were a generalized distortion, then they should show a stronger than normal illusion of control in tasks and activities which are unrelated to gambling. Therefore, it seems important to rely on a more indirect methodology which is unrelated to gambling and which can collect indicators of the illusion of control that are not mediated by introspection. For all these reasons, we propose that the study of pathological gamblers should benefit from using the same assessment techniques that are typically used in the study of contingency judgments and illusions of control in general associative learning theory and research. Of particular interest, from our point of view, is that this methodology will allow us to test, not whether gamblers develop illusions of control during gambling, but, most importantly, whether they tend to overestimate cause-effect relationships in other areas of their life as well.

## Contingency learning and the illusion of control

The clinical and social psychology approach to the illusion of control has typically explained this illusion as a means to protect self-esteem (e.g., Taylor and Brown, 1988; Alloy and Clements, 1992). However, these illusions have also been reported in many cases in which participants are not personally involved and their self-esteem is not at risk, as when participants ask somebody else to roll a dice for them (e.g., Wohl and Enzle, 2009), when participants are just spectators in a sports competition and believe they influence their team's results (e.g., Pronin et al., 2006), or when participants develop the illusion by just observing or being told that someone took a (fake) medicine and reported feeling better (Matute et al., 2011). Associative learning researchers have explained the illusion of control as a special case of the illusion of causality, a cognitive bias that takes place in most people when associating causes and effects in null contingency situations (Matute et al., 2011). In this framework, being personally involved or trying to protect self-esteem is not critical, as the illusion is thought to be the output of the way our cognitive system interacts with the world and extracts contingency and causal information from it (e.g., Matute, 1996; Msetfi et al., 2005, 2007; Allan et al., 2008; Matute et al., 2011).

In order to infer that a causal relationship exists, the potential cause (the participants' action, in the case of the illusion of control) and the outcome should be contingent to each other. A commonly used index of contingency is the Δp index (Jenkins and Ward, 1965; Allan and Jenkins, 1983). It is calculated as the probability of the outcome occurring when the potential cause (i.e., the response, in the case of illusion of control) has been presented P(O|C), minus the probability of the outcome occurring when the cause is absent, P(O|¬ C). That is, Δp = P(O|C) − P(O|¬ C).

A zero contingency relationship between our behavior and an outcome would be that in which the outcome occurs with the same probability regardless of whether we perform the response. Thus, a value of Δp of 0 means that our behavior does not cause the outcome. An illusion of control is said to occur in a zero contingency situation whenever people report a subjective judgment of contingency significantly higher than 0. This is a very common illusion of causality which has been shown in many different experiments in the associative learning literature (e.g., Alloy and Abramson, 1979; Wasserman et al., 1983; Matute, 1996; Allan et al., 2005; Msetfi et al., 2005, 2007; Matute et al., 2007; Hannah and Beneteau, 2009; Blanco et al., 2012). According to associative theories, this illusion is a consequence of the associative learning mechanism constantly trying to associate causes and effects. It sometimes overestimates the relationship between potential causes and effects, particularly under certain conditions.

One of the variables that has been most clearly established to affect the development of the illusion of causality is the probability of the outcome, for instance, the probability with which spontaneous remissions of pain occur (e.g., Alloy and Abramson, 1979; Allan and Jenkins, 1983; Matute, 1995; Wasserman et al., 1996; Buehner et al., 2003; Allan et al., 2005, 2008; Msetfi et al., 2005, 2007; Musca et al., 2010). Another variable that is known to affect this illusion is the probability of responding (or, more generally, the probability with which the potential cause occurs; e.g., Allan and Jenkins, 1983; Matute, 1996; Wasserman et al., 1996; Perales et al., 2005; Hannah and Beneteau, 2009; Matute et al., 2011; Vadillo et al., 2011). The higher these two probabilities, the higher the probability that coincidences will occur between the potential cause and the outcome, and thus, the higher the probability than an illusion of control will develop (see Blanco et al., 2011, 2013; Hannah and Beneteau, 2009).

Therefore, we used a standard task that measures perceived contingency with respect to an actual null contingency in a fictitious medical scenario. The outcome was programmed to occur at high rate (i.e., high frequency of spontaneous recovery in fictitious patients), so that control participants would develop the illusion, particularly if they responded frequently. This procedure should be low on biases inherent to introspective and domain-specific measures, but should nevertheless induce an illusion of control in most participants. If pathological gamblers suffer from a stronger-than-normal distortion in their general perception of contingency, their bias should manifest in this standard medical judgments task as compared to the control group. If this were the case, this would mean that the gamblers' misperception of control is not restricted to their gambling activities, but could possibly be generalizable to other aspects of their daily life.

## Methods

### Participants and apparatus

One hundred anonymous participants took part in this experiment. The gambler group was recruited through FEJAR (Spanish Federation of Rehabilitated Gamblers). It consisted of 49 participants (42 men and 7 women, mean age = 40.4, SD = 11.31) who had been diagnosed of pathological gambling using the South Oak Gambling Screen Questionnaire (i.e., SOGS, see Leiseur and Blume, 1987, Spanish adaptation by Echeburua et al., 1994). They were currently in rehabilitation stage. Their voluntary and anonymous participation was requested through FEJAR. The experiment was available during 6 months at our online laboratory, http://www.labpsico.deusto.es, so that participants in both groups could access the experiment at their convenience.

The control group consisted of 51 anonymous Internet users (27 men and 24 women, mean age = 37.04, *SD* = 10.54) who happened to visit our online laboratory (because they were visiting a web site or social network that linked our laboratory or because they were searching the Internet for concepts related to information published in our laboratory, or because of other reasons) during the time the experiment was available, and voluntarily decided to participate. To increase participation and following ethical standards for human research over the Internet (Frankel and Siang, 1999), we never ask participants in our online laboratory to provide additional personal or demographic data, nor do we use cookies or software to obtain information without their consent.

Internet experiments could be in principle suspect to providing noisy data, but they have been shown to yield results that are as reliable as those observed in the laboratory if certain cautionary measures are taken (e.g., Kraut et al., 2004; Germine et al., 2012; Ryan et al., 2013). Most importantly for our present purposes, illusion of control effects have already been replicated both in the laboratory and through the Internet using associative learning procedures similar to the one we are using here (e.g., Matute et al., 2007; Blanco et al., 2013).

### Procedure and design

Participants performed a task known as the “Contingency Judgments Task,” which, under different variations and versions, is frequently used in the study of associative learning (e.g., Allan et al., 2005; Msetfi et al., 2005; Blanco et al., 2011). In our procedure, participants were asked to imagine being a medical doctor who was using an experimental medicine, Batatrim, which might cure painful crises produced by a fictitious disease called Lindsay Syndrome. They were also told that the effectiveness of Batatrim had not been proven yet and that this medicine produced some secondary effects, so that they needed to use it with caution (this instruction was given so that participants would not administer Batatrim at every opportunity to their fictitious patients). Participants were exposed to the records of 100 fictitious patients suffering from Lindsay's crises, one patient per trial. In each trial, the screen was divided in three horizontal panels. In the upper panel, participants were informed that that patient was suffering a crisis. In the second panel, participants could choose between giving or not giving Batatrim to this particular patient. Responses to this question were given by clicking on one of two bottoms, “Yes” or “No.” The lower panel of each trial was presented immediately after participants entered their response. It showed whether the fictitious patient overcame the crisis. It also showed a “click to continue” bottom that participants could click at their pace in order to continue to the next trial.

After the 100 training trials, participants were asked to rate the efficacy of Batatrim in healing the crises. For this purpose the following question was presented in the middle of the screen: to what extent do you believe that Batatrim has been effective in healing the crises of the patients you have seen?” This test question was answered in a scale ranging from 0 (labeled “Definitely not”) to 100 (labeled “Definitely”).

The outcome (healings) occurred with a probability of 0.80, but following a pseudorandom order which was independent of the participants' behavior. As mentioned in the Introduction, the reason we are using a high probability of the outcome is because this has been shown to favor the development of the illusion in most people in previous reports (Alloy and Abramson, 1979; Allan and Jenkins, 1983; Matute, 1995; Hannah and Beneteau, 2009). Thus, even though it occurred very frequently, the outcome was absolutely independent of the participants' behavior, which means that any subjective estimation of control that is significantly greater than 0 can be considered an illusion of control. Most importantly, the critical question of this experiment is whether the gambler group will show a stronger illusion than the control group under this high-outcome procedure.

## Results

As could be expected from previous reports on the illusion of control using a high probability of the outcome, both groups of participants overestimated the contingency between their behavior and the outcome. Student's *t*-tests confirmed that in both groups the judgments of contingency were significantly higher than 0, *t*_(48)_ = 24.42, *p* < 0.01 for the gamblers group, and *t*_(50)_ = 13.85, *p* < 0.01 for the control group. The critical result in this experiment, however, is the stronger illusion of control that was observed in the gamblers group (*M* = 70.61, SE = 2.89) as compared to the control group (*M* = 57.20, SE = 4.12). A *t*-test revealed that this difference was statistically significant, *t*_(98)_ = 2.643, *p* = 0.010. These data indicate that pathological gamblers perceived a stronger illusory relationship between their behavior and the desired outcome in a medical diagnostic task commonly used to assess associative learning and contingency judgments in laboratory settings.

In addition, and in line with previous reports (Matute, 1996; Blanco et al., 2009, 2011; Hannah and Beneteau, 2009), the results of this experiment showed a significant correlation between the probability with which participants administered the medicine to their fictitious patients and their judgment of control, Pearson's *r* = 0.418, *p* < 0.01. That is, the higher the probability of responding, the higher the illusion of control. Interestingly, however, there were no significant differences in the probability with which the gamblers group (*M* = 63.27, SE = 0.054) and the control group (*M* = 59.69, SE = 0.048) administered the medicine to their patients, *t*_(98)_ = 0.492, *p* > 0.05. Thus, as expected, participants' judgments of control were highly correlated with their probability of responding, so that they developed stronger illusions as responding increased. However, the higher illusion observed in the gamblers group was not due to stronger responding in this group. Thus, a genuine difference in the way they process causal information seems to be responsible for the stronger illusion shown by this group.

Before we finish this section some comment is in order in relation to the possible influence of demographic variables such as age and gender on the observed results. First, no significant differences were observed between the two groups with respect to age, *t*_(98)_ = 1.373, *p* > 0.05, thus, the observed differences cannot be attributed to this variable. However, and despite the experiment being available online during 6 months, the gamblers group was composed mainly of men. Thus, the effect of gender cannot be properly analyzed. Nevertheless, the results of the present experiment are exactly opposite to what should be expected if the effect of group and gender had been confounded. Previous research had shown that women are more vulnerable to the illusion of control than men (Alloy and Abramson, 1979; Wong, 1982; Vyse, 1997; Wolfradt, 1997; Dag, 1999). Thus, if anything, a group composed mainly of men should have shown a weaker, rather than a stronger illusion.

## Discussion

The present results show that in a standard medical judgmental task in which the outcome occurs at a high rate and most people develop an illusion of control, pathological gamblers show an illusion that is even stronger than that of control participants. That is, in this experiment, the actual causal relationship between the participants' administering a medicine to the fictitious patients and the healing of the patients was non-existent, but even so, gambler participants perceived it as highly contingent (compared to a control group without diagnosed pathology who also developed the illusion but less intensely). The use of this associative learning task, which also reflects a feasible situation in daily life (i.e., using medication to reduce pain or illness), suggests that the illusion of control may be a generalized problem in gamblers' daily life and is, therefore, not restricted to their gambling behavior. This has implications for our understanding of the way pathological gamblers process causal information outside of the gambling domain, and may provide hints for a more general assessment and treatment of their problem.

As previously mentioned, the illusion of control is explained from most associative learning theories as a misperception of contingencies that takes place in most people under certain conditions. There are some differences between different theories, and this misperception could occur through several different mechanisms. For instance, it could be due to people giving more weight to cases that confirm that their behavior is followed by the desired outcome and less weight to other information such as, for example, those cases in which the result occurs when they do not act (e.g., cases in which the health crises are also overcome even when the patient is not given the medicine). Several associative theories have contemplated a weighted Δp rule, in which people would weight differently the different types of information that can be encountered, with maximal weight given to those cases in which both the potential cause and the outcome are present, and minimum weight to cases in which neither one is present (e.g., Wasserman et al., 1996). A related but slightly different approach has been taken by associative theories that emphasize the differential perception of contextual information or, in other words, the way participants perceive what happens during the time in which no cues or outcomes are being presented and therefore they are just exposed to the experimental context (Msetfi et al., 2005, 2007). There are also theories that propose that the locus of the distortion does not reside at the perception (or encoding) stage, but at the subsequent judgmental stage (e.g., Allan et al., 2008). Yet, other theories have emphasized the role of the probability of responding (or, more generally, of the potential cause), so that, for instance, participants have been shown to expose themselves to more (adventitious) cause-effect coincidences when they respond frequently to obtain the outcome (i.e., assuming the outcome also occurs with high frequency, which is usually the case in situations in which illusions occur; see e.g., Matute, 1996; Hannah and Beneteau, 2009; Matute et al., 2011; Blanco et al., 2012, 2013). In these cases the number of accidental coincidences increases as the probability of the cause increases (e.g., as participants respond more), thereby the illusory perception of causality becomes stronger as well. The locus of the illusion of control here is therefore behavioral: the more participants respond, the greater their illusion.

This latter view is the one we have favored in many previous reports, and the basic finding that the probability of responding influences the illusion has been replicated in many experiments (e.g., Matute, 1996; Blanco et al., 2009, 2011, 2012; Hannah and Beneteau, 2009). The general effect of the probability of responding has also been replicated in the present experiment, in which the outcome was frequent and the results showed that the higher the probability of responding, the higher the illusion of control. Importantly, however, the probability-of-response effect was clearly not responsible for the stronger illusion of control developed by the gamblers group, as differences in response probability were not observed between the two groups. Thus, even though the present experiment was not designed to discriminate among the different theories of the illusion of control, it seems clear that the locus of the stronger illusion observed in the gamblers group in this medical task resides, not at the behavioral level, but at the perceptual or the judgmental stages.

The present results also suggest that the development of programs and strategies to help people be more accurate in their general detection of contingencies could be a good complement to clinical therapies designed to eliminate gambling behavior. Cognitive interventions for pathological gambling usually focus on cognitive behavioral therapy (Gooding and Tarrier, 2009) and on identifying and restructuring cognitive distortions (Ledgerwood and Petry, 2005; Fortune and Goodie, 2011). Making use of strategies developed under the general associative learning framework to reduce the likelihood of overestimation of contingencies could probably be a helpful addition. Indeed, proper training in recognizing the actual relationships between actions and outcomes in different non-gambling situations could possibly help patients learn to detect the lack of control in situations where there is no contingency between the events, at least in non-gambling conditions (see e.g., Wasserman et al., 1983; Matute, 1996; Msetfi et al., 2005, 2007; Hannah and Beneteau, 2009; Matute et al., 2011; Blanco et al., 2012).

As mentioned in the Introduction, many experiments have shown that a high outcome probability favors the development of the illusion of control. This outcome density effect was documented in the 1980's and 1990's (Alloy and Abramson, 1979; Allan and Jenkins, 1983; Matute, 1995) and is still a topic of high relevance in the experimental study of contingency learning (Buehner et al., 2003; Allan et al., 2008, 2005). For this reason, we used a high-outcome schedule. Control participants should develop the illusion and in this way a potentially stronger illusion could be observed in the gamblers group when confronting this standardized procedure. Thus, it is important to note that the present research does not speak to the issue of how the illusion of control operates during the low outcome conditions which are common during gambling. As many authors have already stated, other factors are critical in explaining the origin and maintenance of gambling behavior, such as, for instance, variable schedules of reinforcement (Ferster and Skinner, 1957) and the fact that gamblers often mention that their gambling behavior was reinforced during the early trials (Molde et al., 2009). Our research is silent with respect to those factors and to gambling behavior itself. What it shows is that gamblers are more vulnerable to the illusion of control than control participants in other areas of their life.

Our findings raise several questions about the role the illusion of control plays in pathological gambling (Myrseth et al., 2010). It is possible that people who are more vulnerable to the illusion of control have a greater risk of falling into gambling behavior, though it might also be that it is gambling behavior what increases vulnerability to the illusion of control. In this respect, the experimental assessment of the illusion of control that we propose can provide a richer and more complete assessment in different situations and could serve therefore as predictor or detector of the appearance of the pathology. Longitudinal studies could therefore be of use in future research to test this view.

On the limitation side of our experiment, the fact that the gamblers group was composed mostly of men could be problematic. Despite our leaving the experiment online for 6 months we were unable to obtain more female gamblers to participate in the study. This might reflect, on the one hand, their lower proportion in the general population (Desai et al., 2005; Blanco et al., 2006), and on the other one, their greater reluctance to publicly acknowledge and discuss their condition, perhaps because gambling has been traditionally regarded as a male activity (Potenza et al., 2001). Thus, given the asymmetrical distribution of participants we were unable to analyze the effect of gender on the observed results. Nevertheless, previous research had shown that men tend to show weaker illusions of control than women (Alloy and Abramson, 1979; Wong, 1982; Vyse, 1997; Wolfradt, 1997; Dag, 1999), which suggests that, if anything, our gamblers group should have shown a weaker, rather than a stronger, illusion than the control group, had the results been confounded by gender. In any case, it will be necessary to achieve better control of this variable in future research.

Another potential problem is that we did not ask our participants to provide any demographic or personal information in addition to their age and gender. We always do it this way in order to increase participation and to comply with ethical standards on anonymity and privacy in our online experiments. However, because in the current experiment group assignment was not random, it might have occurred that the two groups differed by chance in some particular variable, such as, for instance, number of years of formal education, and this might have affected the development of the illusion of control. We believe this is unlikely, and reviews on related effects, such as superstitious beliefs, have concluded that there are no consistent results on the effects of variables such as years of education, or even general intelligence, on the development of these types of biased thinking (Wiseman and Watt, 2006). Nevertheless, it would also be desirable to obtain information on a larger number of demographic variables in future experiments.

To sum up, we believe that the use of the medical associative learning task may be an appropriate way to measure the illusion of control, not only in the general population but also in people with pathology such as gambling, which seems to be especially vulnerable to this type of illusion. One advantage of the associative learning task that we used is that it can easily detect the illusion of control in more general conditions and life areas, not necessarily related to pathology. Moreover, this procedure provides a less clinical perspective and is more focused on general associative-learning skills, so that the biases it detects are, at least in principle, domain-independent and common to most people, though some people are more vulnerable than others. This should allow researchers and therapists to use the large amount of already published evidence on contingency learning to test new and innovative strategies to reduce these biases in pathological gamblers (and other) populations under clinical treatment.

### Conflict of interest statement

The authors declare that the research was conducted in the absence of any commercial or financial relationships that could be construed as a potential conflict of interest.
